# The Atacama Desert: A Biodiversity Hotspot and Not Just a Mineral-Rich Region

**DOI:** 10.3389/fmicb.2022.812842

**Published:** 2022-02-09

**Authors:** Benito Gómez-Silva, Ramón Alberto Batista-García

**Affiliations:** ^1^Laboratory of Biochemistry, Biomedical Department, Health Sciences Faculty and Centre for Biotechnology and Bioengineering (CeBiB), Universidad de Antofagasta, Antofagasta, Chile; ^2^Centro de Investigación en Dinámica Celular, Instituto de Investigación en Ciencias Básicas y Aplicadas, Universidad Autónoma del Estado de Morelos, Cuernavaca, Mexico

**Keywords:** The Atacama, biodiversity, extreme environments, extremophiles, lithobionts, new paradigm

## Introduction

The Atacama Desert in northern Chile is a coastal nonpolar hyperarid desert with nearly 1,000 km long located in South America (latitudes 19°S and 30°S), between the Pacific Ocean to the west and the Andes Range to the east (Bull et al., 2016). It is also considered the oldest and driest desert on Earth (Houston and Hartley, 2003; McKay et al., 2003; Sun et al., 2010). Historically, the Atacama Desert has been described as a barren, desolate, lifeless, harsh environment for life with an undisputed mineral richness under exploitation since pre-Columbian times (Philippi, 1860; OPSAL, 2021). A solid and growing body of information on microbial and genetic richness of the Atacama Desert has been published during the first two decades of the twenty-first century. It sustains that this hyperarid region is no longer just a mineral-rich and sterile territory and must be conceptually redefined to include its biological resources. This integral view of the Atacama Desert ecosystem should raise environmental, social, and educational impacts as well as scientific and technological progress in the region. The reports cited in this contribution are a selection from a large body of substantial and informative articles on the microbiology of the Atacama.

## Bases for a New View of the Atacama Desert

**Figure 1:**
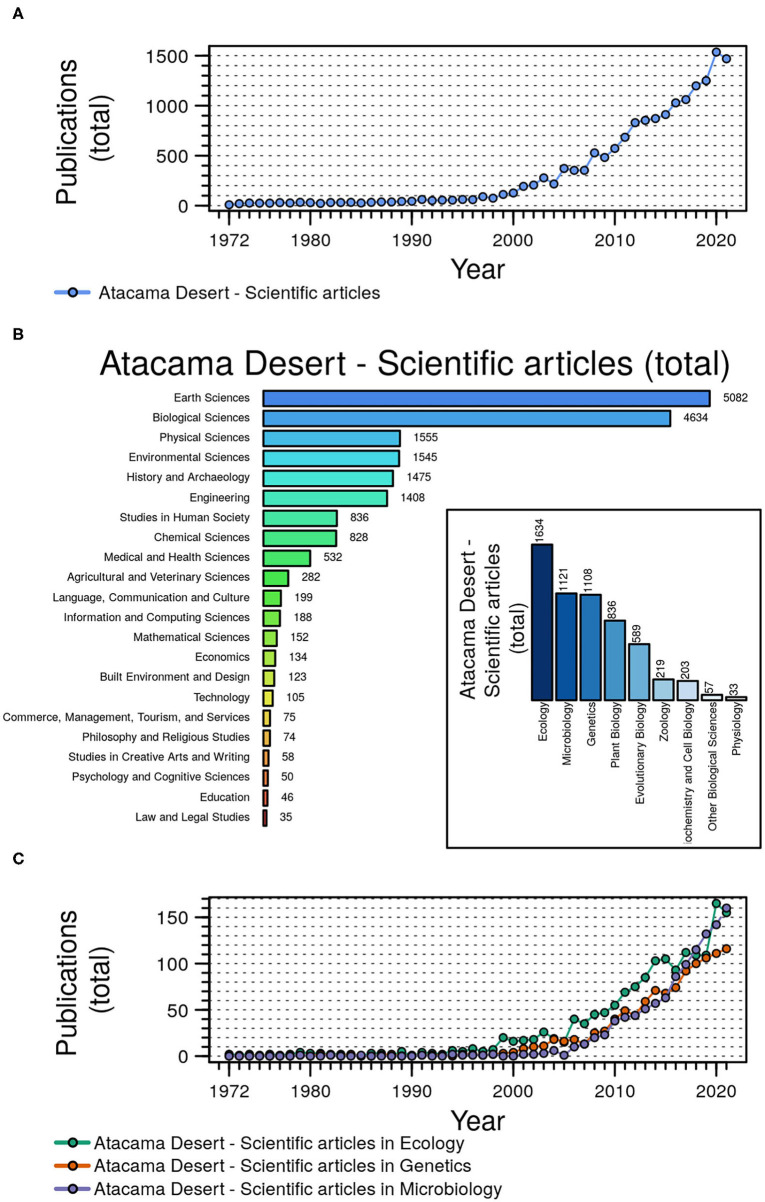
Dynamic of the scientific article production about the Atacama Desert in the period 1972–2021. **(A)** Publication of scientific articles about the Atacama Desert by year. **(B)** Distribution of the number of scientific publications about the Atacama Desert in different research categories. The inserted panel shows the number of publications in different subcategories belonging to biological sciences. **(C)** Scientific publication on the Atacama Desert by year in three different subcategories: Ecology, Microbiology, and Genetics. Figure was prepared in R environment (R base). Data of publications was collected from https://app.dimensions.ai on November 07, 2021.

Articles published by the mid-1960s and at the beginning of the present century (Cameron et al., 1966; Dose et al., 2001; McKay et al., 2003; Navarro-González et al., 2003) have been considered seminal and a driving impulse for a substantial number of microbiology-related reports on the Atacama Desert. Figure 1 provides a comprehensive and quantitative overview on the number and types of scientific publications on different disciplines on the Atacama since 1972, particularly on ecology, genetics, and microbiological studies conducted during the present century.

Life in our planet proliferates in almost any habitat known to have available liquid water sources. Abundance and diversity of life forms are limited by the prevailing physical and chemical variables in the Atacama Desert, as well as in other extreme environments on Earth (Rothschild and Mancinelli, 2001; Gómez-Silva, 2010; Bull et al., 2016; Meslier et al., 2018). High desiccation (aridity index near or below 0.05) and one of the highest solar insolation in our planet (UV index of 15–20) are the two major environmental factors limiting life in the Atacama and highly restrictive to microorganisms without the appropriate strategies to cope with them (Houston and Hartley, 2003; McKay et al., 2003; Cordero et al., 2014; Bull et al., 2016; Gómez-Silva, 2018; Meslier et al., 2018). Not surprisingly, the Atacama has a relatively abundant microbial population at wetter habitats such as wetlands and non-fossil salars (salt flats with surface and/or underground liquid water inflows from rains and rivers, e.g., Salar de Atacama and Salar de Llamara). Many articles have shown the experience of research groups in isolation, culturing, taxonomy, metabolic capabilities, genomic studies and, biochemical characterization of individual microorganisms and microbial consortia from the Atacama (Cabrol et al., 2007; Rainey et al., 2007; Dorador et al., 2010; Gramain et al., 2011; Farías et al., 2014; Bull et al., 2016; Finstad et al., 2017; Castro et al., 2018; Santiago et al., 2018; Warren-Rhodes et al., 2019; Flores et al., 2020; Galetovic et al., 2020; Salazar-Ardiles et al., 2020; Shen et al., 2021a; Vignale et al., 2021; Villalobos et al., 2021; among many other important reports). Microbial research has also been focused on the Atacama's habitats without any regular source of liquid water, such as soils, rocks, and fossil salars. Lithic substrates are proper refuges for microbial colonization in the absence of regular supply of liquid water, a fact with evident astrobiological implications (Wierzchos et al., 2006; Gómez-Silva, 2010, 2018; Gramain et al., 2011; Robinson et al., 2015; Finstad et al., 2017; Meslier et al., 2018). Extensive metagenomics studies have demonstrated that lithobiontic life in the Atacama includes members of the three domains of life and viruses (Gómez-Silva et al., 2019; Uritskiy et al., 2019; Hwang et al., 2021). Hipolithic, endolithic, or epilithic colonization by cyanobacteria-dominated microbial consortia have been reported in quartz, halites and gypsum substrates, where the microbial communities harvest liquid water from atmospheric water vapor by salt deliquescence (Davila et al., 2008), capillary condensation (Wierzchos et al., 2012), and fog droplets (Azúa-Bustos et al., 2011).

Bioprospection and analyses of microbiological, metagenomics and other studies on novel extremophiles and extreme-tolerant microorganisms from the Atacama have opened new biotechnological opportunities based on the search of novel secondary metabolites, genes, gene clusters, metabolic pathways, peptides, pigments, and macromolecules, with impacts in biomedicine, food and feed supplements, biological control and other activities (Rateb et al., 2011; Gonçalves et al., 2015; Gómez-Silva et al., 2019; Flores et al., 2020; Galetovic et al., 2020; Salazar-Ardiles et al., 2020).

Twenty years of active research in the Atacama have provided substantial microbiological information; however, additional studies are required to properly assess the role of the microbial community structure in ecosystem functioning, provisioning, and supporting services. Chemolithotroph life in the Atacama is an example of pending issues to be addressed in future studies focused on its ecological value. Metagenomics studies on the halite microbiome in the Atacama have shown a limited genetic repository for sulfate oxidation but the presence of genes of all the enzymes involved in the assimilatory sulfate reduction pathway, with direct implication on sulfate uptake from oceanic fog, amino acid biosynthesis, microbial metabolism, and sulfur cycle (Gómez-Silva, 2010; Gómez-Silva et al., 2019). Also, active metabolic capabilities such as photosynthetic and transcriptional activities have been demonstrated in microbial consortia inhabiting halites and wetlands, and nitrogen cycling along the rainfall gradient in the Atacama (Davila et al., 2015; Orellana et al., 2020; Uritskiy et al., 2020; Castro-Severyn et al., 2021; Shen et al., 2021b). New questions and ingenious experimental approaches are needed to produce comprehensive knowledge about the role of microbes in biogeochemical cycles, the cellular bases that assist the crosstalk between microbial cells, the trans-kingdom molecular mechanisms playing key roles in the gene expression dynamic, among other topics not covered to date. Undoubtedly, the answers to these and other questions will decisively contribute to the understanding of the ecological role of microbial life in the Atacama.

Although not commented in depth in this document, the Atacama Desert harbors a unique vegetation with many endemic lineages and endangered species, mostly restricted to coastal fog oases or lomas formations and at pre-Andes and Andes ranges. These members of the Atacama's biodiversity have adapted their life cycles to selective sites along this hyperdesert and, locations and identification of Cactaceae and other vascular plants in the Atacama have been acknowledged in molecular and botanical reports but also by an ancestral savvy (Rundel et al., 1991; Ruhm et al., 2020; Eshel et al., 2021). The Atacama plants are essential elements of the Atacama scenery and its natural beauty but also for biochemical research and biotechnological applications. Regulations against illegal sampling of plants, seeds and microbes for scientific and commercial purposes are urgently needed.

## Discussion

*The Atacama Desert is a lifeless territory, worth only for the exploitation of its mineral content*. This summary notion, still present worldwide, supports massive extractive activities with evident and assessable environmental damages, minimal positive cultural impacts but ethno-cultural fractures in the local communities. Centuries-old natural hydrogeological processes have generated underground and surface water bodies on the Salar de Atacama, a pre-Andean endorheic basin with an impressive diversity of life forms adapted to salinity, high solar radiation, and daily and seasonal temperature changes, with important nesting and feeding sites. Also, underground brines with high salts content (lithium and others) are today pumped to evaporation ponds with serious impact on the water content of aquifers and on biodiversity (OPSAL, 2021). It is interesting to contrast this limited conception of the Atacama with the ethnographic and anthropological evidence that trace human settlements in the Atacama territory back to the end of the Pleistocene to the present days. The inherited ancestral knowledge has allowed generations of the Atacama's inhabitants to live in close communion with the biological resources of this extreme region, in a respectful and intelligent practice, particularly with the underground and surface water sources (Philippi, 1860; Núñez et al., 2010; Babidge, 2015; Rivera et al., 2018).

Based on the Atacama biological richness, the proposal of an alternative and conceptually broader vision of this extreme dryland is upheld today by activities associated to the use of renewable energy sources (solar and wind), seawater desalination, and tourism for special interests (mountains, lakes, ponds, vegetation, and wetlands). The Atacama is also a natural laboratory that has and will provide solid advances in paleontology, archaeology, anthropology, astronomy, astrobiology, and other disciplines. It seems evident and necessary that a “new Atacama Desert” be recognized as a desert biome containing mineral richness, highly diverse extant life forms, renewable energy resources, geographic, oceanic, and astronomical assets, and inhabitants with an inherited ancestral acknowledge. New commercial ventures on its natural resources must be carefully regulated to avoid, not only diminishing, their environmental impacts. The biological resources of the Atacama biome, as well as the effects on living organisms and human communities, have not and are not properly protected today by the Chilean legislation; more information can be obtained from documents associated to the Nagoya Treaty (https://www.cbd.int/countries/?country=cl), Babidge (2015) and OPSAL (2021). The intrinsic value of the Atacama biodiversity needs urgent management and protection by proper and pending regulatory policies. Members of the microbial life inhabiting sites with commercially extractive interests in the Atacama (salars, land, mountains, and water sources) are particularly endangered since “*they cannot be seen*” as easily as plants and animals. Today, the microbiome colonizing extreme habitats in the Atacama Desert is invaluable evidence on the evolution of life in our planet; they might not have “c*ommercial*” value but represent scientific and cultural assets subjected to irreversible loss.

Social and natural sciences are contributing to a better understanding of this hyperarid land and the universe around us. The Atacama Desert is no longer the same has we have previously learned to know. It would be expected that this new perspective of “*a new Atacama Desert*,” if broadly accepted, be included in educational plans for new generations of children, researchers, and be shared with the not-scientific society, in Chile and abroad.

## Author Contributions

BG-S and RB-G were responsible of all the content expressed in this Opinion article. All authors contributed to the article and approved the submitted version.

## Funding

This work was supported by CONICYT grant CeBiB FB-0001 (Chile) and CONACYT grant 1559 (Mexico).

## Conflict of Interest

The authors declare that the research was conducted in the absence of any commercial or financial relationships that could be construed as a potential conflict of interest.

## Publisher's Note

All claims expressed in this article are solely those of the authors and do not necessarily represent those of their affiliated organizations, or those of the publisher, the editors and the reviewers. Any product that may be evaluated in this article, or claim that may be made by its manufacturer, is not guaranteed or endorsed by the publisher.
